# Can our microbiome break our hearts? Collaborative production of *p*-cresol sulfate and indoxyl sulfate by commensal microbes increases susceptibility to thrombosis

**DOI:** 10.1128/mbio.02692-23

**Published:** 2024-01-16

**Authors:** Tamara R. Machado Ribeiro, Camila B. Brito, Mariana X. Byndloss

**Affiliations:** 1 Department of Pathology, Microbiology, and Immunology, Vanderbilt University Medical Center, Nashville, Tennessee, USA; 2 Howard Hughes Medical Institute, Vanderbilt University Medical Center, Nashville, Tennessee, USA; University of Hawaii at Manoa, Honolulu, Hawaii, USA; Cleveland Clinic, Cleveland, Ohio, USA

**Keywords:** microbiota, metabolites, thrombosis

## Abstract

A recent study published in *mBio* by Nemet et al. revealed the critical role played by two gut microbiota members in producing the metabolites indoxyl sulfate (IS) and *p-*cresol sulfate (*p*CS) (I. Nemet, M. Funabashi,X. S. Li, M. Dwidar, et al., 2023, mBio 14:e01331-23, https://doi.org/10.1128/mbio.01331-23). Understanding microbial pathways leading to IS and *p*CS production is crucial because they are connected to a pre-thrombotic profile, and having high levels of these metabolites increases the risk of cardiovascular diseases (CVD). Hence, this study can offer vital insights into assessing the risk for CVD and identifying potential treatment targets for this disease.

## COMMENTARY

Emerging studies indicate that metabolites produced by gut microbiota impact cardiovascular health. However, the mechanisms by which microbiota-derived metabolites affect cardiovascular disease (CVD), the leading cause of death worldwide (1, 2), are largely unknown. Thus, it is crucial to gain a deeper understanding of the metabolic pathways of gut microbes involved in the biosynthesis of metabolites associated with CVD. This knowledge could lead to discoveries that would aid in managing heart health, preventing the onset of CVD, and developing new microbiota-focused treatment strategies for heart disease and related complications. Specifically, the uremic solutes *p*-cresol sulfate (*p*CS) and indoxyl sulfate (IS) have been implicated in mediating adverse health effects in patients with renal disease. These two compounds possess comparable protein binding properties, exhibit similar elimination rates through dialysis, and share pro-inflammatory characteristics. Both *p*CS and IS are gut microbiome metabolites derived from amino acids previously associated with CVD risks in the setting of impaired kidney function; however, the mechanism by which gut microbiota members produce these metabolites remains to be elucidated (3, 4).

In a study published in *mBio*, Nemet et al. focus on understanding which gut microbes and microbial genes are involved in producing precursor molecules for IS and *p*CS (5). The authors demonstrated that higher levels of the metabolites IS and *p*CS are associated with CVD in patients with renal failure, contributing to an increased risk of mortality, even in the absence of renal impairment or cardiovascular risk factors. The work by Nemet and colleagues also revealed an important connection between microbial metabolic pathways and the host in synthesizing these metabolites. To do so, the authors used two bacterial strains, *Blautia hydrogenotrophica* DSM 10507 and *Clostridium* sp. D5, both containing the *hpdBCA* operon, which encodes subunits of 4-HPA decarboxylase to convert 4-HPA and, to a lesser extent, tyrosine into *p*CS. However, using *in vitro* approaches, the authors discovered that *B. hydrogenotrophica and Clostridium* sp. D5 bacteria were able to convert 4-OH-phenylpyruvic acid (4-HPA) into *p*CS but not tyrosine. This finding led them to hypothesize that the production of *p*CS might result from a collaboration between two types of microbes in the gut. They demonstrated that the first microbe converts tyrosine into a compound marked as 4-HPA, which is then decarboxylated into *p*CS by the second microbe. This process is made possible due to the crosstalk between metabolites produced by the intestinal microbiota. Using a combination of microbial genetics and co-culture experiments, Nemet and colleagues demonstrated that *Bacteroides thetaiotaomicron* converts tyrosine to 4-HPA and *Clostridium* sp. D5 converts 4-HPA to *p*-cresol. Furthermore, *B. thetaiotaomicron* was found to possess the *BT0430* gene for 4-HPA production. The research findings shed light on the involvement of specific gut microbiota members in the synthesis of IS and *p*CS, adding to the existing understanding of the host’s production of these metabolites.

**Fig 1:**
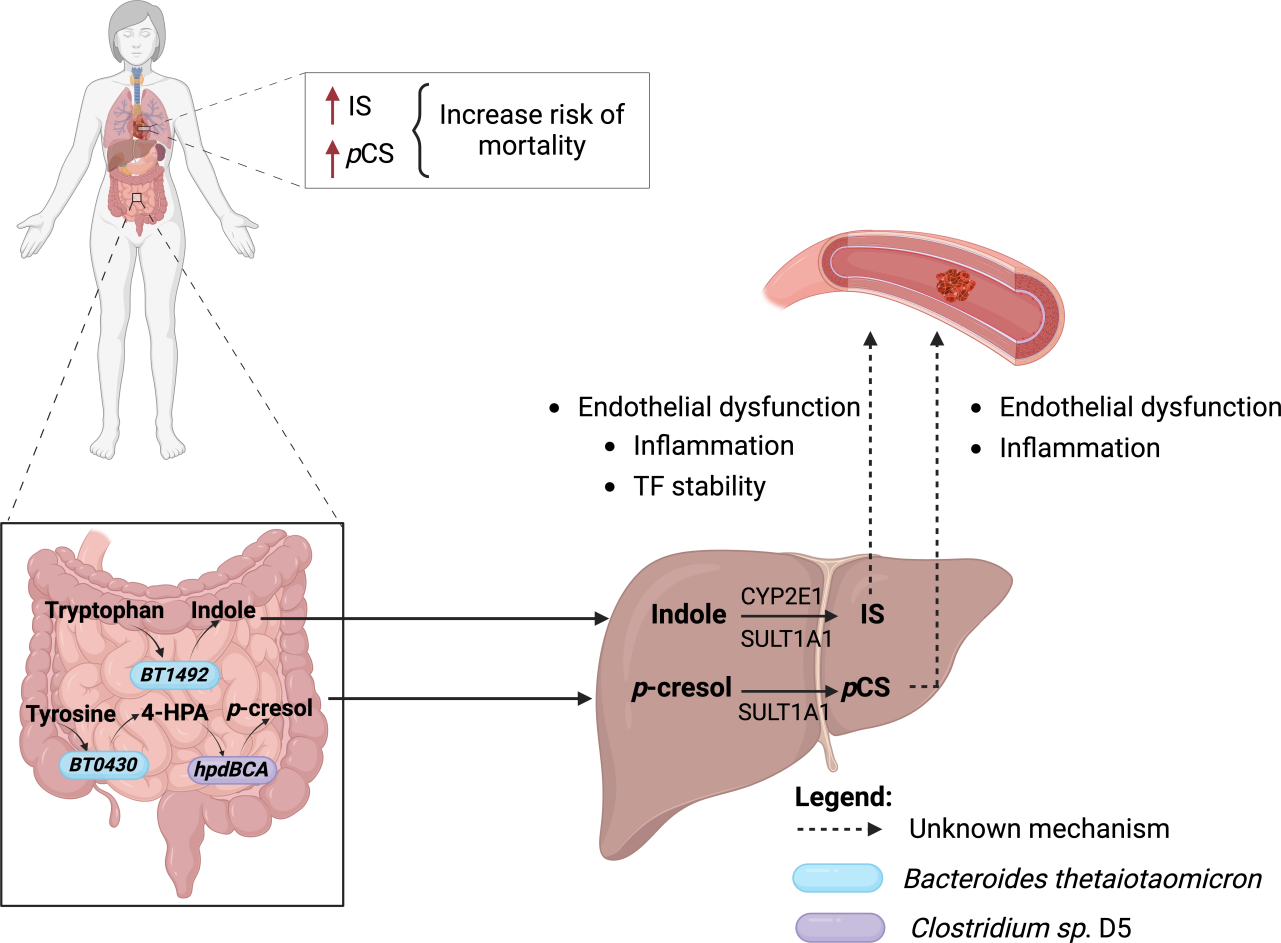
Collaboration between microbiota and host in producing pro-thrombotic metabolites. Increased indoxyl sulfate and *p*-cresol sulfate lead to an increased risk of death in patients with CVD. Tryptophan is metabolized by tryptophanase encoded by *BT1492* gene and found in *Bacteroides thetaiotaomicron*, to indole. Indole is further metabolized by the hepatic enzymes CYP2E1 and SULT1A1 to form IS. Similarly, tyrosine is metabolized to 4-OH-phenylpyruvic acid (4-HPA) by the oxidoreductase, which is encoded by *BT0430* gene and found in *Bacteroides thetaiotaomicron*. Then, 4-HPA is metabolized into *p*-cresol by the hpdBCA operon, which encodes a multi-component 4-hydroxyphenylacetate (4-HPA) decarboxylase. This operon is found in members of the Firmicutes group (here represented by *Clostridium* sp. D5). *p*-cresol reaches the hepatic circulation, where it is converted into *p*CS by the SULT 1A1 enzyme. Finally, *p*CS and IS collaborate individually to increase thrombosis in mice in an unknown mechanism.

The results from *in vitro* studies led the authors to use a genetically modified B. *thetaiotaomicron* VPI-5482 to study the effects of IS, *p*CS, or both metabolites *in vivo* (Fig. 1). They tested whether engineered strains of *B. thetaiotaomicron* contribute to *p*CS and IS production and promote a pro-thrombotic phenotype in the mammalian host, given their relation to the thrombosis phenotype. It is worth noting that both metabolites did not synergize in increasing the pro-thrombotic phenotype, providing evidence of their independent role in thrombosis development (Fig. 1). Finally, analysis of feces from a cohort study revealed that high levels of tryptophanase and *hpdBCA* genes correlate with atherosclerotic cardiovascular disease. This highlights the importance of *p*-CS and indoxyl sulfate in the pathogenesis of CVD.

In conclusion, Nemet et al. elegantly demonstrate the mechanism by which the gut microbiota and the host collaborate in generating the essential amino acid-derived metabolites *p*CS and IS and the role of these metabolites in the CVD. However, one question remains: how do *p*CS and indoxyl sulfate metabolites participate in the predisposition to thrombus formation? Tryptophan metabolites, including indoxyl sulfate, are known to activate the AHR receptor (aryl hydrocarbon receptor) (6). Interestingly, AHR has been demonstrated to play an essential role in tissue factor stability, the initiator of extrinsic coagulation (7). Thus, it would be critical to explore the role of AHR in the predisposition of thrombosis. Furthermore, as discussed by the authors, IS and *p*CS have pro-inflammatory and endothelial dysfunction functions, which could induce an increased risk for thrombosis. Therefore, future studies will be essential to elucidate this mechanism and demonstrate how metabolites from the microbiota can predispose the host to cardiovascular diseases and together contribute to the development of tools for the treatment of CVD.
